# The clinical relevance and prediction efficacy from therapy of tumor microenvironment related signature score in colorectal cancer

**DOI:** 10.3389/fonc.2023.1123455

**Published:** 2023-05-10

**Authors:** Xiang Jun, Shengnan Gao, Lei Yu, Guiyu Wang

**Affiliations:** ^1^ Department of Colorectal Surgery, The Second Affiliated Hospital of Harbin Medical University, Harbin, China; ^2^ Department of Endocrinology and Metabolism, The Second Affiliated Hospital of Harbin Medical University, Harbin, China

**Keywords:** CRC, therapy, response, TMERSS, immune cell, prediction

## Abstract

**Introduction:**

As the top 3 cancer in terms of incidence and mortality, the first-line treatment for CRC includes FOLFOX, FOLFIRI, Cetuximab or immunotherapy. However, the drug sensitivity of patients to regimens is different. There has been increasing evidence that immune components of TME can affect the sensitivity of patients to drugs. Therefore, it is necessary to define novo molecular subtypes of CRC based on TME immune components, and screen patients who are sensitive to the treatments, to make personalized therapy possible.

**Methods:**

We analyzed the expression profiles and 197 TME-related signatures of 1775 patients using ssGSEA, univariate Cox proportional risk model and LASSO-Cox regression model, and defined a novo molecular subtype (TMERSS) of CRC. Simultaneously, we compared the clinicopathological factors, antitumor immune activity, immune cell abundance and differences of cell states in different TMERSS subtypes. In addition, patients sensitive to the therapy were screened out by correlation analysis between TMERSS subtypes and drug responses.

**Results:**

Compared with low TMERSS subtype, high TMERSS subtype has a better outcome, which may be associated to higher abundance of antitumor immune cell in high TMERSS subtype. Our findings suggested that the high TMERSS subtype may have a higher proportion of respondents to Cetuximab agent and immunotherapy, while the low TMERSS subtype may be more suitable for treatment with FOLFOX and FOLFIRI regimens.

**Discussion:**

In conclusion, the TMERSS model may provide a partial reference for the prognosis evaluation of patients, the prediction of drug sensitivity, and the implementation of clinical decision-making.

## Introduction

Colorectal cancer (CRC) represents the third most common malignancy and the second leading cause of cancer death worldwide (1, 2). In recent years, radical resection has been the mainstay of treatment for CRC. In order to avoid recurrence and prolong OS, neoadjuvant or adjuvant chemotherapy is often required for surgical patients. Fluorouracil-based combination chemotherapy is recommended for all patients with stage II or III (3). As first-line agents, fluorouracil-based combination chemotherapy includes FOLFOX, CapeOX, and FOLFIRI (4). However, the occurrence of resistance often makes patients benefit less in the course of treatment (5). Studies have shown that adjuvant chemotherapy improves survival rate by only 3% in patients with stage II CRC, and increases by 15% to 20% for stage III CRC (6). Therefore, it is necessary to screen out patients who have good response to fluorouracil-based combination chemotherapy, making personalized treatment possible.

Colorectal cancer has a complex pathogenesis, and many potential factors have an important impact on the occurrence and development of colorectal cancer. Currently, some studies have reported some factors that affect the occurrence and development of colorectal cancer. These include changes in the cellular microenvironment associated with growth and development (7), the microenvironment in which tumors occur, and the impact of gastrointestinal tumors and tumors outside the gastrointestinal tract, such as colon cancer (8), lung cancer (9), and prostate cancers (10, 11). Meanwhile, increasing evidence demonstrates that the tumor microenvironment (TME) plays a crucial role in tumorigenesis and tumor progression (12). The primary composition of TME includes infiltrating immune cells, mesenchymal cells, and extracellular matrix (13). The infiltrating immune cells are composed of multiple immune cell types, such as T cells, macrophages, and neutrophils (14). Various tumor-infiltrating immune cells make TME a double-edged sword, exhibiting an ability to either arrest or support malignancy (15). The complex role of TME makes it possible to classify cancer immunologically in terms of prognosis, chemotherapy, and immunotherapy response prediction. For example, microsatellite instability tumors show a high abundance of Th1 cells, and effector memory T cells, and have a favorable prognosis. Given that TME plays an indispensable role in chemotherapy and immunotherapy resistance (16), we used the gene expression profiles to define novo molecular classifications of CRC based on signatures of various immune components in order to distinguish between drug sensitivity and TME.

In order to define novel molecular classifications of CRC, gene expression profiles of 1775 patients were analyzed. In this study, our main work included: (1) Constructing a scoring model and redefining the molecular classifications of CRC; (2) Identifying TME differences between CRC and screening patients who respond to chemotherapy or immunotherapy, in order to provide the reference for individualized treatment of patients; (3) Evaluating the relationship between molecular classifications of CRC and clinicopathological factors.

## Materials and methods

### Data downloading and processing

The gene expression profile and clinical data of patients were obtained from GEO, TCGA, and cBioportal databases. The datasets obtained from GEO include GSE17538, GSE12945, GSE39582, and GSE103479. RNA-seq data were collected from the TCGA (https://portal.gdc.cancer.gov/) for 33 cancers. Meanwhile, RNA-seq data of CRC also were collected in cBioportal (https://www.cbioportal.org/).

We used datasets GSE17538, GSE12945, GSE39582, and GSE103479 as discovery cohorts, and used the ComBat function to remove potential multicenter batch effects between different experiments. CRC data from TCGA and cBioportal databases were used as testing cohorts 1 and 2, respectively. Simultaneously, all the data is integrated as the testing cohort 3.

In this research, we conducted systematic bioinformatics analysis on gene expression profile data of 1775 CRC specimens. In the discovery cohort, 1022 patients from four datasets were included in the study. The specific information of each dataset is as follows: The gene expression profile of tumor tissue samples from 62 patients in GSE12945 dataset; The GSE17538 dataset stores gene expression profiles of 244 specimens, of which 238 gene expression profiles from human CRC tissue samples were used for further analysis; The gene expression profiles of 156 patients in GSE103479 dataset were included in the study; The GSE39582 dataset collected the gene expression profile of 585 samples, including 566 colorectal tumor tissue samples and 19 colorectal normal tissue specimens, of which 566 tumor tissue samples were included in the study. The testing cohort 1 integrates the information of 521 colon cancer samples and 177 rectal cancer samples in TCGA database. After excluding 51 normal tissue samples, the gene expression profile of 647 patients was used for bioinformatics analysis. The testing cohort 2 is RNA-seq data from 106 CRC patients in the cBioportal database. The gene expression profile of all cohorts is integrated in cohort 3, including the gene expression profile of 1775 samples. Finally, we also collected the gene expression profiles of 33 cancers in TCGA database, and >1000,0 samples were used for pan-cancer related analysis.

We also collected gene expression profiles of patients with different treatment regimens, such as GSE104645, GSE72970, GSE78220, GSE91061, and IMvigor210 (17). In the dataset GSE104645, the chemotherapy scheme of 104 patients is FOLFOX, who was used for bioinformatics analysis; In the dataset GSE72970, the chemotherapy scheme of 87 patients is FOLFIRI, who was used for bioinformatics analysis; GSE78220 which includes 28 patients is a dataset on anti-PD1 inhibitor immunotherapy in melanoma; GSE91061 which includes 109 patients is a dataset on anti-PD1 and anti-CTLA4 inhibitor immunotherapy for melanoma; IMvigor210 which includes 348 patients is the dataset of anti-PDL1 inhibitor immunotherapy for patients with urothelial carcinoma.

### Collection of TME related signatures

Through an extensive online literature search, we screened 197 representative TME-related signatures from diverse resources. Among them, 68 signatures come from the work of Wolf et al. (18), 25 signatures were from the work of Bindea et al. (19), 24 signatures were obtained from Miao et al.’s work results (20) and 17 signatures were obtained from the Import database (21). In addition, it also includes some marker genes of immune cells, such as marker genes of 22 immune cells in CIBERSORT (22), marker genes of 10 immune cells in MCP-Counter (23), marker genes of 10 immune cells in the Imsig database (24), and 20 signatures of immune cells recognized by TITR et al. (25). Finally, we also included the marker genes of exhausted CD8^+^T cells (26). More detailed information is listed in the **Supplementary Tables S1, S2**.

### Differential expression analysis and enrichment analysis

The differential expression analysis of the data is performed by the R package “limma”. In this study, the threshold value is |log2FC|>1 and FDR<0.05.

We performed a single sample gene set enrichment analysis (ssGSEA) based on the gsva function to assess the infiltration level of signatures in each sample. The normalized enrichment scores (NESs) generated by ssGSEA are regarded as the infiltration level of signatures.

The enrichment analysis of GO and KEGG (27) is achieved by the R package “clusterProfiler”. Meanwhile, we also used KEGG, gendoo, gene2pubmed and Reactome databases for gene set enrichment analysis (GSEA).

### Construction of TME related signature score model

We used the discovery cohort for ssGSEA to calculate the NESs. Then, the NESs was used to construct a univariate Cox proportional hazard model for 197 signatures. And 129 signatures were determined significantly related to the OS (P<0.05).

To screen the most relevant signatures for CRC prognosis in the discovery cohort, the R package “glmnet” were used to construct the LASSO-Cox regression model for 129 signatures. 23 signatures with nonzero coefficients were included in the study, which is the best λ value generated by 10-fold cross validation.

Finally, the hazard ratio (HR) generated by the univariate Cox proportional hazard model was multiplied with the NESs of 23 signatures to construct the TME-related signature score (TMERSS). The calculation formula is as follows:


$$TMERSS=\sum_{i=1}^{n}log(HRi)*NESi$$

HR_i_ is the HR of the i^th^ TME related signature, and NES_i_ is the NES of the i^th^ TME related signature, n=23.

### Calculating the proportion of immune cells and cell states

We quantified the proportion of immune cells in samples by CIBERSORT, MCP-Counter, xCell, and quanTIseq. In order to have a more comprehensive understanding of the state and functional patterns of different immune cells, we based on EcoTyper (https://ecotyper.stanford.edu/) calculating dominant cell states in each sample and the cell states abundance.

### NTP analysis and filtering of signatures

The NTP classification tool (28) is used to calculate the classification of each sample in a specific signature. The signature list of CRC pathologic phenotypes and drug-related genes obtained from previous studies is as follows: intestinal stem cell signature (29), colon crypt signature (30), serrated CRC signature (31), EMT signature (32), FOLFIRI response signature (33), FOLFOX response signature (34) and VEFG/EGFRi signatures (35) described by Schutte et al., including Avastin, Cetuximab, Afatinib, Sapitinib, Gefitinib and Vandetanib.

### Cell lines and qRT-PCR

Human CRC 5-FU sensitive/resistant cell line HCT8/HCT8-5FU and Cetuximab sensitive/resistant cell line Caco2/Caco2-CTX were purchased from Shanghai Meixuan Company (Shanghai, China) and cultured according to previous reports (36).

According to the manufacturer’s instructions, total RNA was extracted and reverse transcribed using TRIzol reagents (Invitrogen, Carlsbad, CA, USA) and cDNA reverse transcription kits (Applied Biosystems, Foster City, CA). SYBR Green reagent (Thermo Fisher Scientific, Waltham, MA) was used for qRT-PCR experiments. With β-action is an internal parameter that is passed through 2^-ΔΔCT^ method calculate the relative expression of the target gene. The primer sequence information is shown in **Supplementary Table S3**.

### Western blot and CCK-8 assay

Western blot analysis was performed to determine the protein expression levels of LAMB1, APOC1, and AREG. The protein was extracted by SDS-PAGE and transferred to the PVDF membrane. They were incubated overnight with anti LAMB1 (1:1000, Cell Signaling Technology, 4723S), APOC1 (1:1000, Cell Signaling Technology, 3957S), AREG (1:1000, Cell Signaling Technology, 8751), and GAPDH (ZSGB-Bio, TA-8) primary antibodies at 4°C. After incubation with horseradish peroxidase linked secondary antibodies for 2 hours, ECL (Beyotime, China) was used to visualize the signal.

Cells were implanted in 96 well microplates and administered 10 μg/ml of 5-FU, 200 μg/ml Cetuximab intervention for 24, 48, or 72 hours. Add 10 μl of CCK-8 solution (Dojindo) to each well, incubate at 37°C for 2 hours, and measure the OD value at 450 nm.

### Statistical analysis

We used the R package “survminer” to calculate the optimal cut-off value. Meanwhile, Kaplan-Meier survival curves of patients with different subtypes were plotted based on R package “survminer” and “Survival”. We divided patients into four consensus molecular subtypes (CMS) by using the R package “CMScaller”.

In this study, all statistical analyses were conducted based on the R programming language. All statistical tests are two-sided, and P< 0.05 is considered statistically significant.

## Results

### Establishment of a scoring model based on TME related signatures

**Figure 1:**
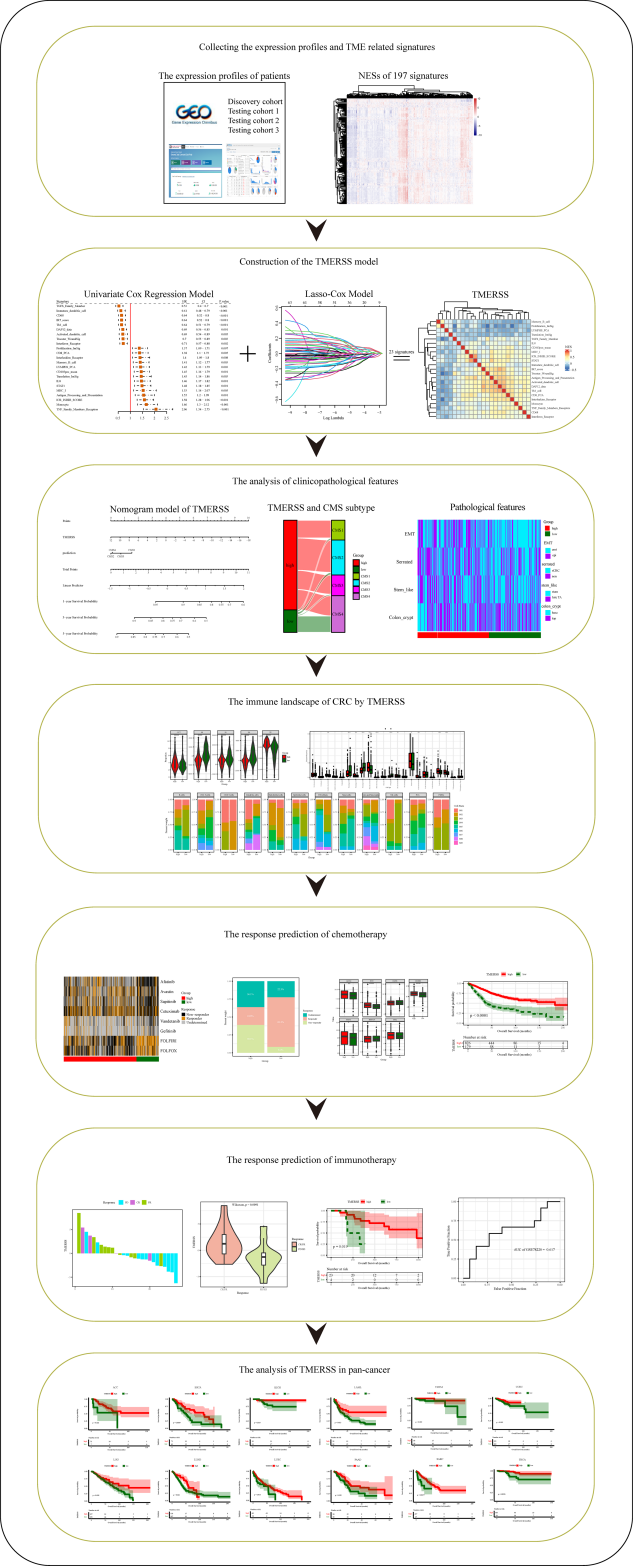
Schematic diagram of the study design. *p-value < 0.05, **p-value < 0.01, ***p-value < 0.001, ****p-value < 0.0001.

**Figure 2:**
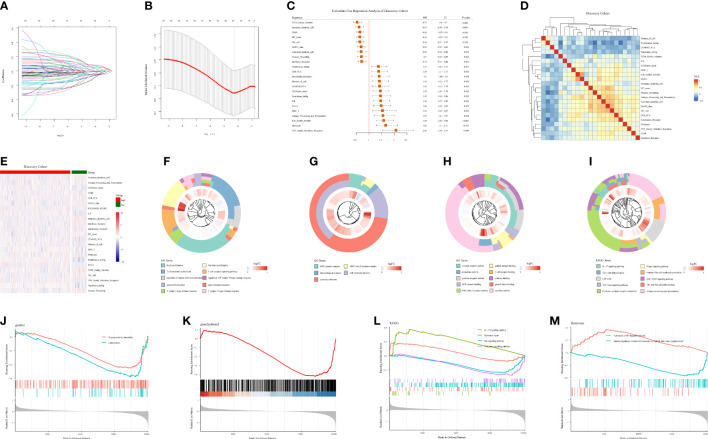
Building the TMERSS model by the discovery cohort. **(A)** LASSO coefficient distribution of 129 signatures; **(B)** LASSO regression model showed partial likelihood deviation in 10-fold across validation; **(C)** The forest of 23 signatures; **(D)** The heatmap of spearman’s correlation between 23 signatures; **(E)** The heatmap according to NESs of 23 signatures; **(F–H)** Visualization of 10 terms in BP, CC and MF, respectively; **(I)** 10 KEGG pathways of differentially expressed genes in distinct TMERSS subtypes; **(J–M)** The databases gendoo, gene2pubmed, KEGG and Reactome were used for GSEA of TMERSS model related genes, and the terms associated with TMERSS was described.

The design of this study is exhibited in **Figure 1**. Based on TME-related signatures, we conducted ssGSEA on all samples to calculate NESs. After initial screening, 192 signatures were obtained that were present in all cohorts. First, univariate Cox proportional hazard regression analysis was conducted on 192 signatures in the discovery cohort. We found that 129 signatures were significantly related to the OS of patients (P<0.05). Subsequently, LASSO-Cox regression models were used to screen for signatures highly associated with outcomes. In this model, lambda.1se=0.06028869 (**Figures 2A, B**), and the results show that the coefficients of 23 variables are nonzero. The relationship between infiltration level and survival of 23 signatures is shown in the forest (**Figure 2C**). By calculating the correlation coefficients among 23 signatures (**Figure 2D**), we found that there are mainly three types of relationships among signatures. Namely, negative correlation (Memory_B_cell, Proliferation_ImSig, LYMPHS_PCA and Translation_ImSig), positive correlation (IR7_score, Troester_WoundSig, Antigen_Processing_and_Presentation, Activated_dendertric_cell, DAP12_data, and Th1_cell) and weak correlation (MHC_I, ICR_INHIB_SCORE, STAT1, Monocyte, and Interleukins_Receptor). The results of the testing cohorts further confirmed the relationships among 23 signatures (**Supplementary Figures 1A-C**). Finally, based on the HRs of 23 signatures and their infiltration levels in each patient, we constructed the TMERSS model.

To analyze the transcriptome and immunology heterogeneity of patients, we calculated the optimal cut-off value based on TMERSS values, divided patients into high and low TMERSS subtypes, and compared the heterogeneity of 23 signatures of immune infiltration levels between the two subtypes. The results showed that there was no significant difference in immune infiltration levels between the two subtypes (**Figure 2E**; **Supplementary Figure 1D–F**).

Furthermore, we conducted an enrichment analysis of 23 signature genes to determine their biological functions. As expected, the enrichment analysis results of these genes are closely related to TME (**Figures 2F-H**). For example, T−helper 17 type immune response and immune receiver activity. The KEGG pathway is enriched to immune and oncogenic related pathways, such as natural killer cell mediated cytotoxicity and JAK−STAT signaling pathway (**Figure 2I**). We also performed GSEA on the gene expression data of two subtypes of patients based on KEGG, gendoo, gene2pubmed, and Reactome databases (**Figures 2J–M**).

### TMERSS is associated with clinicopathological features of colorectal cancer

**Figure 3:**
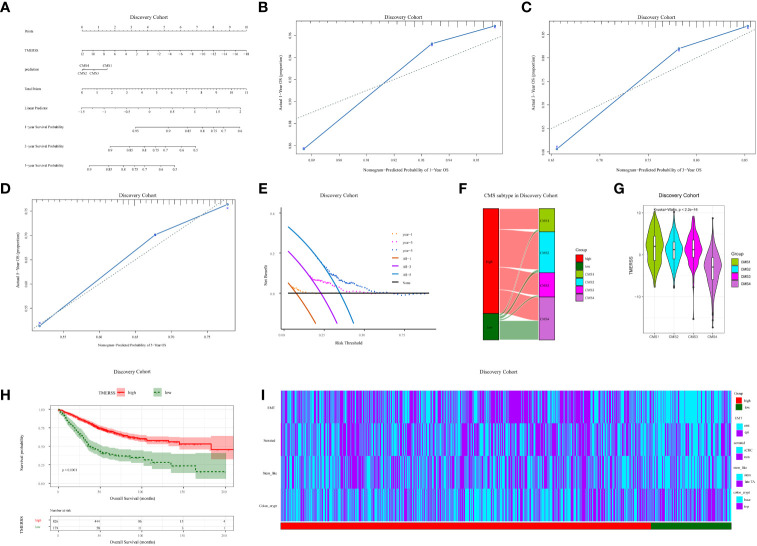
Correlation analysis of clinicopathological factors in the discovery cohort. **(A)** Nomogram, containing TMERSS and CMS; **(B-D)** Observing the consistency between the predicted 1, 3 and 5-year survival probability and the actual survival probability according to calibration curves. The predicted survival probability of nomogram is displayed on the x-axis, and the actual survival probability is displayed on the y-axis. The ideal curve of nomogram is represented by a dotted line along the 45-degree angle; **(E)** Analysis of decision curves for 1, 3, and 5-year, with black lines indicating assuming no patient dies within 1, 3, and 5-years; **(F)** Sankey of TMERSS and CMS subtypes; **(G)** The violin shows the distribution of TMERSS values of different CMS; **(H)** Kaplan-Meier survival curve according to the overall survival of TMERSS subtypes; **(I)** The heatmap of pathological factors of TMERSS subtypes based on published gene signatures.

We further analyzed the relationship between TMERSS and clinicopathological features in four cohorts. A nomogram model containing information about the TMERSS and CMS subtypes was constructed by the discovery cohort (**Figure 3A**). Compared with CMS subtypes, it was evident that TMERSS contributes most of the risk points. Based on nomogram calibration curves, we used TMERSS to predict the 1, 3, and 5-year survival probabilities of patients. The calibration curve of 1-year survival probability cannot perfectly fit the ideal curve (**Figure 3B**), while calibration curves of 3-year and 5-year survival probability can well predict the survival probability of patients (**Figures 3C, D**). Similarly, the decision curve analysis showed that the nomogram was poor at predicting 1-year survival probability because of its low clinical net benefit (**Figure 3E**); Because of the high clinical net benefit in the 3-year and 5-year decision curves, the nomogram can well predict the 3-year and 5-year survival probability (**Figures 3F, G**). Overall, these observations indicated that the nomogram of TMERSS proved well discrimination and calibration capabilities.

In 2015, Sabine et al. divided CRC into CMS1-CMS4 subtypes and analyzed the relationship between each subtype and the prognosis of patients (37). Here, by comparing the relationship between distinct TMERSS and CMS subtypes in the discovery cohort, we found that high TMERSS subtypes are mainly associated with CMS2, while low TMERSS subtypes are associated with CMS4 (**Figures 3H, I**). In testing cohort 1, the high TMERSS subtype was evenly distributed across CMS subtypes, while the low TMERSS subtype was strongly correlated with CMS4. Of course, the results of testing cohorts 2 and 3 were similar to the discovery cohort (**Supplementary Figures 2A, B**). It is well known that among CMS subtypes, CMS4 exhibits poorer OS, while CMS2 exhibits longer OS (37). The Kaplan-Meier survival curve of the study confirmed that the survival probability of the high TMERSS subtype was higher than that of the low TMERSS subtype (**Figure 3H**; **Supplementary Figure 2C**), which was consistent with the survival probability of patients among CMS subtypes.

We used the previously reported gene signatures to identify the cellular and precursor origins of TMERSS subtypes based on the NTP algorithm. Applying the intestinal stem cell signature and colon crypt signature to the expression data of four cohorts (**Figure 3I**; **Supplementary Figure 2D**), we found that low TMERSS subtype significantly enriched the stem-like and colon top crypt phenotype. Considering that epithelial-mesenchymal transition (EMT) plays a crucial role in the development and progression of CRC (38), we used the EMT signature for analysis. The results showed that the low TMERSS subtype significantly enriched the “emt” phenotype, while the high TMERSS subtype more expressed the epi phenotype.

### Heterogeneity of tumor immune response between TMERSS subtypes

We have constructed a TMERSS model based on 23 signatures. Although the infiltration level of 23 signatures has no apparent difference between TMERSS subtypes (**Figure 2E**; **Supplementary Figures 1D–F**), a more systematic characterization and comparison of the heterogeneity of immune responses in their classified samples was still needed. To this end, we summarized the characteristic divergence of TMERSS subtypes from the three aspects of antitumor immune activity, an abundance of tumor-infiltrating immune cells, and functional states of immune cells, and deepened the understanding of CRC classified based on the TMERSS model.

**Figure 4:**
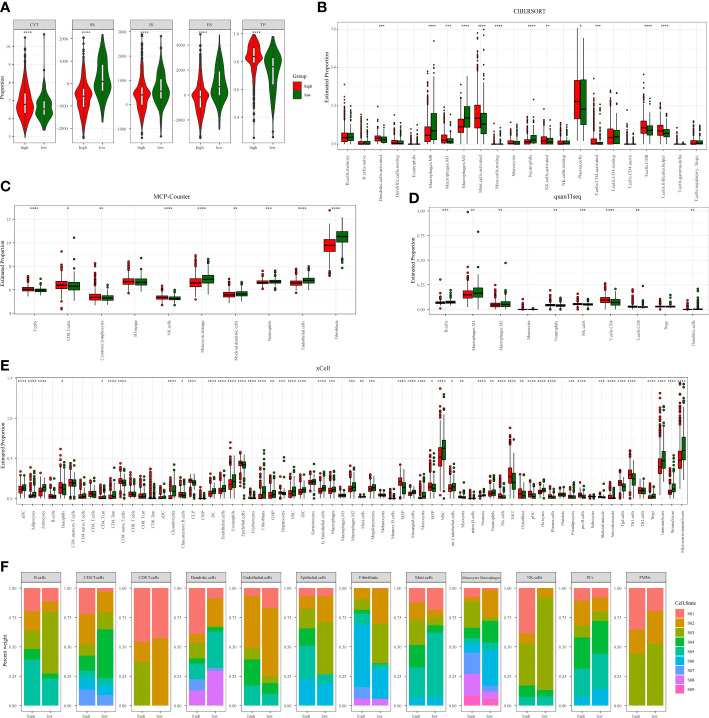
Immunological heterogeneity of TMERSS subtypes in the discovery cohort. **(A)** The differences of CYT, stromal score, immune score, ESTIMATE score and tumor purity among TMERSS subtypes; **(B-E)** Based on CIBERSORT, MCP-Counter, quanTIseq and xCell, the proportion of immune cells between high and low TMERSS subtypes was estimated. ns ≥ 0.05, *< 0.05, **< 0.01, ***< 0.001 and ****< 0.0001; **(F)** Distribution of immune cell states in different TMERSS subtypes.

In combination with the characterization of the immune activity of CRC, we observed the differences between distinct TMERSS subtypes from the level of immune response activity. First, the variation in immune microenvironments of TMERSS subtypes is reflected in the overall level of immune infiltration. We calculated the immune score and stromal score for TME based on ESTIMATE. The results showed that the low TMERSS subtype had the higher immune score and stromal score, but tumor purity was lower than that of the high TMERSS subtype (**Figure 4A**, **Supplementary Figure 3A**). Simultaneously, there was significant variation in the antitumor immune activity of TMERSS subtypes, with high TMERSS subtype having a higher cytolytic activity (CYT).

Then, four deconvolution tools were used to analyze differences in the abundance of tumor-infiltrating immune cells. We observed that antitumor immune cells are highly expressed in high TMERSS subtypes, such as NK cells, cytotoxic T cells, CD8^+^T cells, etc. (**Figures 4B–E**; **Supplementary Figures 3B–E**). Conversely, tumor-promoting immune cells are highly expressed in low TMERSS subtypes, such as cancer-associated fibroblasts, M2 macrophages, dendritic cells, and regulatory T cells.

To have a more comprehensive understanding of the state and functional pattern distinction of cells in different TMERSS subtypes, we determined the dominant cell states and cell state abundance in each sample based on the EcoTyper algorithm and carried out a comparative analysis. In the machine learning framework, EcoTyper, each immune cell is considered to have multiple cell states. Such as CD8^+^T cells have 3 cell states (Naïve/central memory (S01), Late-stage differentiated effector (S02), and Exhausted/effector memory (S03)), epithelial cells have 6 cell states (Basal-like (S01), Normal-enriched (S02), Pro-angiogenic (S03), Pro-inflammatory (S04), Unknown (S05), and Metabolic (S06)), mast cells have 6 cell states (Normal-enriched (S01), Normal-enriched (S02), Unknown (S03), Classical (S04), Unknown (S05), and Activated (S06)), dendritic cells have 8 cell states (Myeloid cDC1 (S01), Myeloid cDC2-B (Inflammatory) (S02), Mature immunogenic (S03), Unknown (S04), Mature (normal-enriched) (S05), Langerhans-like (S06), Migratory activated (S07), and Unknown (S08)) and NK cells have 5 cell states (Classical (S01), Normal-enriched (S02), Unknown (S03), Unknown (S04), and Unknown (S05)). The different cell states of more immune cells can be found in **Supplementary Table S4**. We observed significant differences in the proportional distribution of cell states between TMERSS subtypes (**Figure 4F**; **Supplementary Figure 3F**). Some cell states were dominant in the high TMERSS subtype with high immune activity (the proportion is significantly highest), while they are significantly reduced or almost absent in low TMERSS subtype (the proportion is almost 0). For example, the relative proportion of CD8^+^T cells in the Exhausted/effector memory (S03) state, epithelial cells in the Pro-inflammatory (S04) state, Mast cells in the Classical (S04) state, dendritic cells in the Myeloid cDC1 (S01) state, and NK cells in the Classical (S01) state in low TMERSS subtype is almost 0.

These results indicated that there is heterogeneity of tumor immune response between TMERSS subtypes, and high TMERSS subtype show higher antitumor immune activity, abundances of antitumor immune cell, and antitumor immune cell states. This may explain the longer OS of high TMERSS subtype.

### TMERSS model has a potential function to evaluate the chemotherapy response

**Figure 5:**
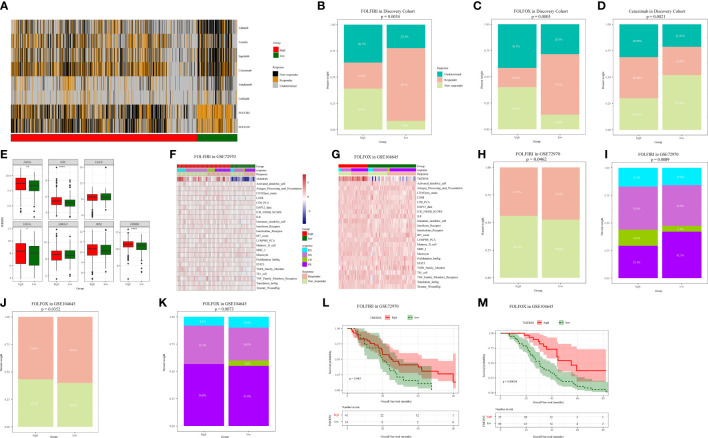
Correlation analysis between TMERSS subtypes and chemotherapy. **(A)** The heatmap of the correlation between the response of single CRC patient to FOLFIRI, FOLFOX and EGFR inhibitors, and the samples with FDR<0.2 were considered significant; **(B-D)** The histogram shows the number of clinical responses of high and low TMERSS subtypes to FOLFIRI, FOLFOX and Cetuximab. Chi-square test p-value differences are shown; **(E)** Boxplot showed differences in the expression of Cetuximab response-related genes in high and low TMERSS subtypes; **(F)** The heatmap of 23 signatures in GSE72970 cohort; **(G)** The heatmap of 23 signatures in GSE104645 cohort; **(H-I)** The histogram shows the number of clinical responses of high and low TMERSS subtypes to FOLFIRI in GSE72970 cohort. Chi-square test p-value differences are shown; **(J-K)** The histogram shows the number of clinical responses of high and low TMERSS subtypes to FOLFOX in GSE104645 cohort. Chi-square test p-value differences are shown; **(L-M)** Kaplan-Meier survival curve based on OS of TMERSS subtypes in GSE72970 and GSE104645 cohort. *p-value < 0.05, **p-value < 0.01, ***p-value < 0.001, ****p-value < 0.0001.

Chemotherapy plays an indispensable role in the treatment of CRC. In order to make the TMERSS model applicable to the clinic, we analyzed differences in response to chemotherapy drugs in CRC between TMERSS subtypes. Based on the NTP algorithm, we applied drug-related signatures to the gene expression profile of four cohorts to predict the response of patients to eight chemotherapy regimens (**Figure 5A**; **Supplementary Figures 4A–C**). In the discovery cohort, the response rates of low TMERSS subtype to FOLFIRI, FOLFOX, and Cetuximab regimens were 69.3%, 57.5%, and 26.82% respectively (**Figures 5B–D**); Contemporary, the response rates of high TMERSS subtype to FOLFIRI, FOLFOX and Cetuximab regimens were 24.8%, 18.4%, and 38.98% respectively. It is obvious that the low TMERSS subtype has a higher response rate to FOLFIRI, and is more resistant to Cetuximab; However, the response rate of the high TMERSS subtype to FOLFIRI and FOLFOX regimen was low, and it was sensitive to Cetuximab. Except that the response rates of low TMERSS subtype in testing cohort 1 to the FOLFIRI were low (**Supplementary Figure 4D**), the analysis results in other testing cohorts are similar to those in the discovery cohort (**Supplementary Figures 4D–F**). To further analyze the reasons for the differences in the response of TMERSS subtypes to different chemotherapy regimens, we have collected FOLFIRI (33), FOLFOX (34), and Cetuximab (35) sensitive related genes in previous literature, and compared the expression of these genes in different TMERSS subtypes. Compared to the high TMERSS subtype, FOLFIRI, and FOLFOX sensitive related genes are highly expressed in the low TMERSS subtype (**Supplementary Figures 5A, B**). The Cetuximab sensitive related genes are highly expressed in the high TMERSS subtype (**Figure 5E**). In addition, we analyzed the expression of related genes in 5-FU sensitive/resistant cell lines HCT8/HCT8-5FU and Cetuximab sensitive/resistant cell lines Caco2/Caco2-CTX. **Supplementary Figures 5C–E** further confirmed our analysis results. After overexpression of LAMB1, APOC1, or AREG in HCT8-5FU and Caco2-CTX cells, we found that HCT8-5FU and Caco2-CTX cells restored their sensitivity to 5-FU and Cetuximab, respectively (**Supplementary Figures 5F, G**). Moreover, we found that overexpression of LAMB1 or APOC1 can reduce the resistance of HCT8-5FU; Overexpression of AREG can reduce the resistance of Caco2-CTX (**Supplementary Figures 5H–I**). Based on the above results, we speculate that the difference in the expression of chemotherapy-related genes in different TMERSS subtypes may explain to some extent the difference in response between TMERSS subtypes to distinct chemotherapy regimens.

In addition, we analyzed the responses of patients to FOLFIRI and FOLFOX based on datasets GSE72970 and GSE104645. We first analyzed the relationship between infiltration levels of 23 signatures and drug response in the TMERSS model. However, the infiltration level of 23 signatures was not significantly correlated with responses of FOLFOX or FOLFIRI (**Figures 5F, G**). Then, we explored whether the TMERSS model based on the datasets GSE72970 and GSE104645 was related to drug response. In GSE72970, the response rates of high and low TMERSS subtypes to FOLFIRI were 43.9% and 47.4%, respectively. Compared with the low TMERSS subtype, the high TMERSS subtype had a lower proportion of drug resistance to FOLFIRI (**Figures 5H, I**). Similarly, in GSE104645, the response rates to FOLFIRI were 56.8% for the high TMERSS subtype and 60.0% for the low TMERSS subtype, and the low TMERSS subtype was more sensitive to FOLFOX (**Figures 5J, K**). Finally, the relationship between TMERSS subtypes and outcomes was clarified. As expected, the OS of the low TMERSS subtype is shorter (**Figures 5L, M**).

In general, these observations demonstrated that TMERSS model may be used as a potential tool to evaluate the response rate of CRC to chemotherapy. Concurrently, TMERSS subtypes can provide a reference for clinicians to use drugs. The low TMERSS subtype is more suitable for FOLFOX or FOLFIRI, while patients with high TMERSS are more sensitive to Cetuximab.

### Immunotherapy benefits were positively correlated with TMERSS values

**Figure 6:**
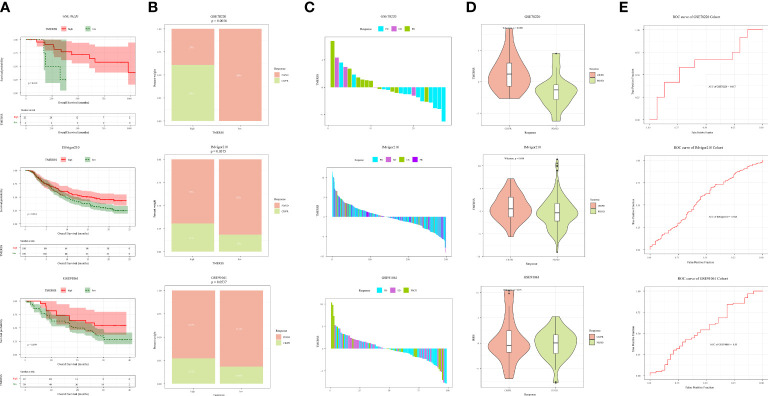
Correlation analysis between TMERSS subtypes and immunotherapy. **(A)** Kaplan-Meier curve according to the OS of TMERSS subtypes in immunotherapy cohort; **(B)** The histogram shows the number of immunotherapeutic responses in the high and low TMERSS subtypes of the immunotherapy cohort. Chi-square test p-value differences are shown. **(C)** The waterfall diagram shows the distribution of patients with different immunotherapeutic response in the immunotherapy cohort; **(D)** The boxplot of TMERSS distribution of patients with different immunotherapy response in immunotherapy cohort; **(E)** ROC curve for predicting response in immunotherapy cohort.

As a novel modality to remedy cancer, immunotherapy has been widely concerned because of the high response rates of cancer patients to immunotherapy. In this research, we wanted to investigate whether the TMERSS model can predict the benefit of immunotherapy in patients. However, after an extensive literature review and extensive literature search, we did not find suitable datasets for CRC immunotherapy, so we explored the relationship between immunotherapy responses and the TMERSS model in the melanoma and uroepithelial carcinoma immunotherapy datasets (GSE78220, GSE91061, and IMvigor210). Kaplan-Meier survival curves showed that the high TMERSS subtype had a better prognosis than the low TMERSS subtype (**Figure 6A**). Across the three immunotherapy datasets, we found that the high TMERSS subtype was more effective in responding to immunotherapy, with higher response rates (**Figures 6B, C**). Compared with progressive-disease (PD)/stable-disease (SD), the violin further confirmed that TMERSS values significantly increased the complete-response (CR)/partial-response (PR) of CRC (**Figure 6D**).

The TMERSS values in three datasets were also evaluated by ROC curves analysis to estimate their predictive potential for immunotherapy benefits. The areas under ROC curves of GSE78220, IMvigor210, and GSE91061 datasets were 0.62, 0.57, and 0.55, respectively (**Figure 6E**), suggesting that the TMERSS model has good predictive efficacy for immunotherapy benefit.

### Analysis of TMERSS model in pan-cancer

**Figure 7:**
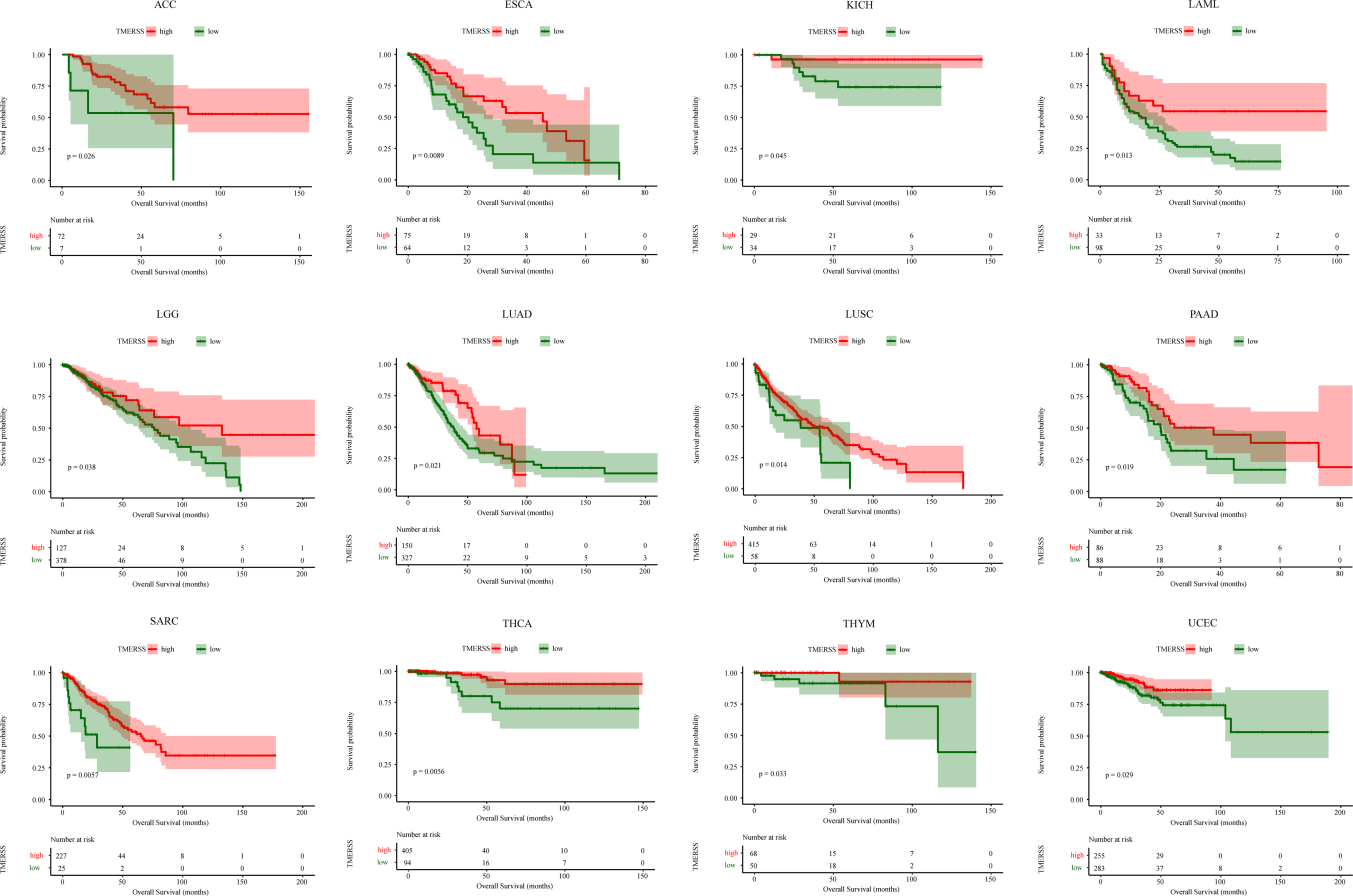
The application of the TMERSS model in pan-cancer. The Kaplan-Meier survival curves of TMERSS subtypes in 12 cancers, which includes ACC (Adrenocortical Carcinoma), ESCA (Esophageal Carcinoma), KICH (Kidney Chromophobe), LAML (Acute Myeloid Leukemia), LGG (Brain Lower Grade Glioma), LUAD (Lung Adenocarcinoma), LUSC (Lung Squamous Cell Carcinoma), PAAD (Pancreatic Adenocarcinoma), SARC (Sarcoma), THCA (Thyroid Carcinoma), THYM (Thymoma) and UCEC (Uterine Corpus Endometrial Carcinoma).

We applied the TMERSS model to other cancers to determine whether it has universal applicability in pan-cancer. Firstly, TPM data of 33 cancers were downloaded from TCGA and TMERSS model was constructed. Then, optimal cut-off points were calculated based on the TMERSS values, and the patients were divided into high and low TMERSS subtypes. Finally, Kaplan-Meier survival curves showed that the prognosis of the high TMERSS subtype was better than that of the low TMERSS subtype in 12 cancers (**Figure 7**). The results confirmed that the TMERSS model may be universally applicable in these cancers.

## Discussion

CRC, like other malignant tumors, is highly heterogeneous (39). The complex interaction between malignant tumor cells and TME contributes greatly to the development and progression of CRC (40). Effective recognition of the distinction of diver immune components in TME may help explain the heterogeneity of CRC. ESMO guideline recommends FOLFIRI and FOLFOX as first-line chemotherapies for metastatic CRC. Although FOLFIRI or FOLFOX can significantly prolong the median OS, nearly 50% of patients cannot benefit from it (41). Therefore, screening patients with potential responses to FOLFOX and FOLFIRI is an urgent priority.

We performed univariate Cox regression analysis on 197 signatures to identify those that are significant for prognosis. Then the optimal 23 variates were selected by the LASSO-Cox regression model, and the TMERSS model was constructed based on 23 signatures. Further analysis showed that the molecular subtype based on the optimal cut-off point could effectively distinguish TME and drug sensitivity.

Firstly, relationships between TMERSS subtypes and clinicopathological factors were analyzed. Of the two TMERSS subtypes, the high TMERSS subtype has a longer OS. The association analysis between TMERSS and CMS subtypes revealed that the majority of low TMERSS subtypes were included in CMS4, and the low TMERSS subtype had similar results with CMS4, that is, the OS was shorter (37); The high TMERSS subtype contains mainly CMS2, and the longer OS of high TMERSS subtype is consistent with the longer OS of CMS2 (37). In addition, the previously reported association analysis between gene signatures and TMERSS subtypes also revealed the potential biological characteristics behind TMERSS subtypes. For example, serrated precursor tumors were significantly associated with the low TMERSS subtype. In the low TMERSS subtype, the stem-like and emt phenotypes were significantly enriched.

The heterogeneity of tumor immune response determines differences in prognosis in different patients, and infiltrating immune cells play a vital role in tumor immune response. NK cells, CD8^+^T cells, and cytotoxic T cells (42) are considered as main antitumor immune cells, while fibroblasts and regulatory T cells promote the occurrence and development of tumors. In our study, the high TMERSS subtype enriched antitumor immune cells, which is consistent with the improvement of prognosis of antitumor immune cells; In contrast, the low TMERSS subtype has a higher abundance of immunosuppressive cells. Our results show that the OS of the low TMERSS subtype is shorter than that of the high TMERSS subtype. Further cell states analysis also found that the immune cell states in the high TMERSS subtype mostly showed antitumor immune activity, while the low TMERSS subtype lacked such cells.

The high heterogeneity of CRC also affects the sensitivity of chemotherapy. Studies have shown that the stem-like phenotype of CRC has a high response rate to FOLFIRI (43), which is consistent with our results, low TMERSS subtypes enrich the stem-like phenotype and are sensitive to FOLFIRI. The response rates of FOLFOX were similar to that of FOLFIRI and were resistant to the high TMERSS subtype. The low TMERSS subtype has shorter OS but is more sensitive to FOLFOX or FOLFIRI, suggesting that the low TMERSS subtype is a potential response to FOLFOX or FOLFIRI and that FOLFOX or FOLFIRI has the potential to improve the prognosis of low TMERSS subtype. In our results, compared with the low TMERSS subtype, the high TMERSS subtype has a higher response rate to Cetuximab, which may be related to the higher expression of Cetuximab responsive-related genes in high TMERSS subtype.

In addition, there is growing evidence that patients with microsatellite instability are sensitive to immune checkpoint inhibitors, and CMS1 was rich in a higher proportion of microsatellite instability (37). In our analysis, CMS1 was mainly associated with a high TMERSS subtype. Based on datasets GSE78220, GSE91061, and IMvigor210, we analyzed the association of TMERSS subtypes with immune response and prognosis of patients. Unlike the low TMERSS subtype, which has high response rates to FOLFOX or FOLFIRI, TMERSS values significantly increased the sensitivity of patients to immune checkpoint inhibitors in immunotherapy cohorts. In short, high TMERSS subtypes are more sensitive to immunotherapy. We also observed that the OS of the high TMERSS subtype was longer than that of the low TMERSS subtype.

This study has several limitations worth acknowledging. First of all, the analysis is based on previously published data, which is a retrospective study, and more real data are needed for prospective analysis and verification; Secondly, due to the incomplete clinical information of data, more clinicopathological factors were not included in the study, such as TNM stage, age, sex, tumor pathological type, etc. Finally, we only divided patients into two subtypes according to the optimal cut-off value, and more classification algorithms need to be explored to further define and classify TMERSS subtypes.

Together, we constructed the TMERSS model by using public datasets and 197 signatures to define novo molecular subtypes. TMERSS subtypes have different effects on the prognosis of patients. Moreover, the TMERSS model reveals the efficiency of chemotherapy or immunotherapy to a certain extent and may be a potential tool for predicting the response of chemotherapy or immunotherapy. The high TMERSS subtype may be more suitable for Cetuximab treatment or immunotherapy, while the low TMERSS subtype may be more sensitive to FOLFIRI or FOLFOX regimens.

## Data availability statement

The datasets presented in this study can be found in online repositories. The names of the repository/repositories and accession number(s) can be found in the article/**Supplementary Material**.

## Ethics statement

All methods were carried out in accordance with relevant guidelines and regulations. Research involving human participants and human data, performed in accordance with the Declaration of Helsinki.

## Author contributions

All authors contributed to the article and approved the submitted version. XJ and LY designed the project. XJ and SG performed administrative, technical, or material support. Jun Xiang performed statistical analysis. XJ wrote the manuscript. GW and LY revised the paper.
