# Supplementary material for: The clinical relevance and prediction efficacy from therapy of tumor microenvironment related signature score in colorectal cancer
Source: Front Oncol. 2023 May 10;13:1123455. doi: 10.3389/fonc.2023.1123455 (PMC10207322; doi:10.3389/fonc.2023.1123455)
Supplement: Supplementary file 1 [file DataSheet_1.docx]

**Supplementary Figures**


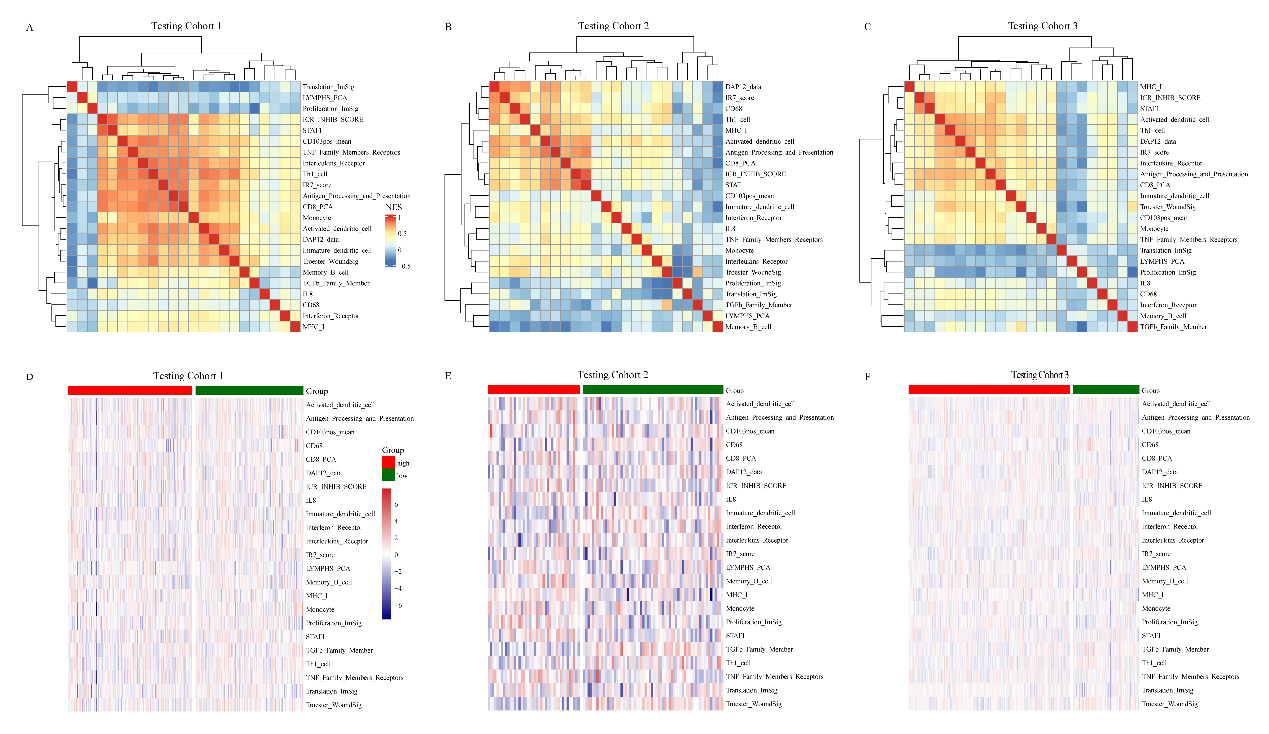


Supplementary Figure 1. Validating the TMERSS model in testing cohorts. (A-C) are spearman correlation coefficient heatmaps of 23 signatures in testing cohort 1, 2 and 3, respectively; (D-F) Drawing the heatmap according to the NESs of 23 signatures in testing cohort 1, 2 and 3, respectively.


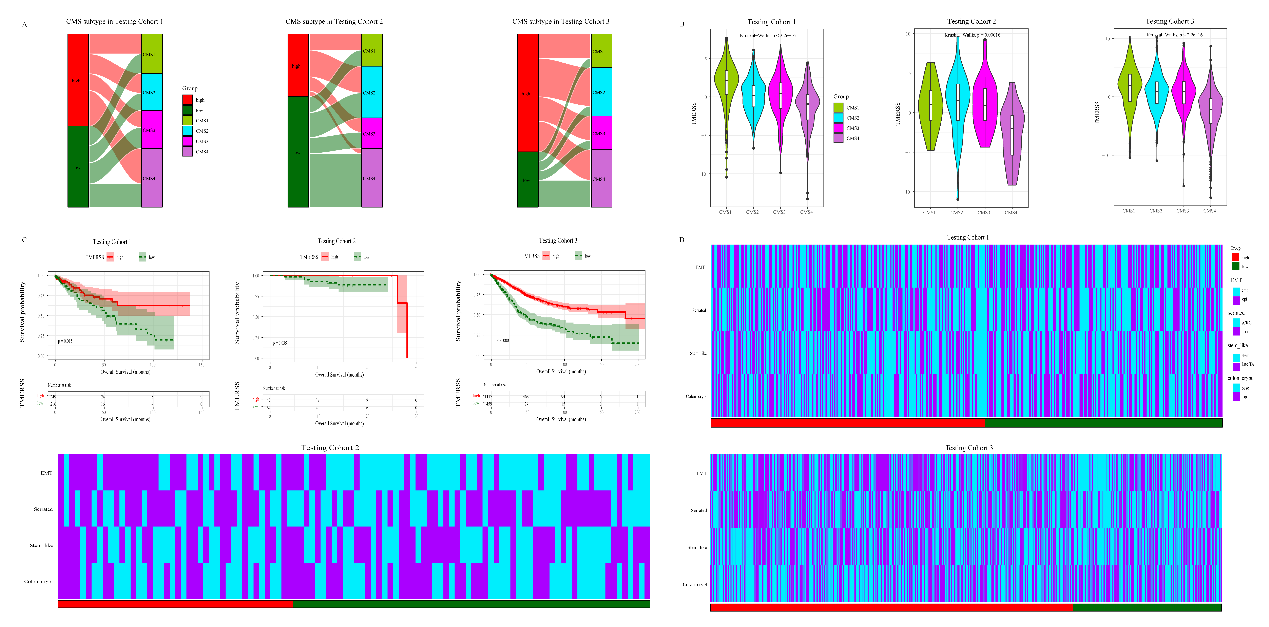


Supplementary Figure 2. Association analysis of clinicopathological factors in testing cohorts. (A) Sankey of the TMERSS subtype with different consensus molecular subtypes in three testing cohorts; (B) The violin shows the distribution of TMERSS values of different consensus molecular subtypes in the three testing cohorts; (C) Kaplan-Meier survival curves based on overall survival of the TMERSS subtype in three testing cohorts; (D) Heatmaps of key pathological factors of the TMERSS subtype based on the signature of published genes in three testing cohorts.


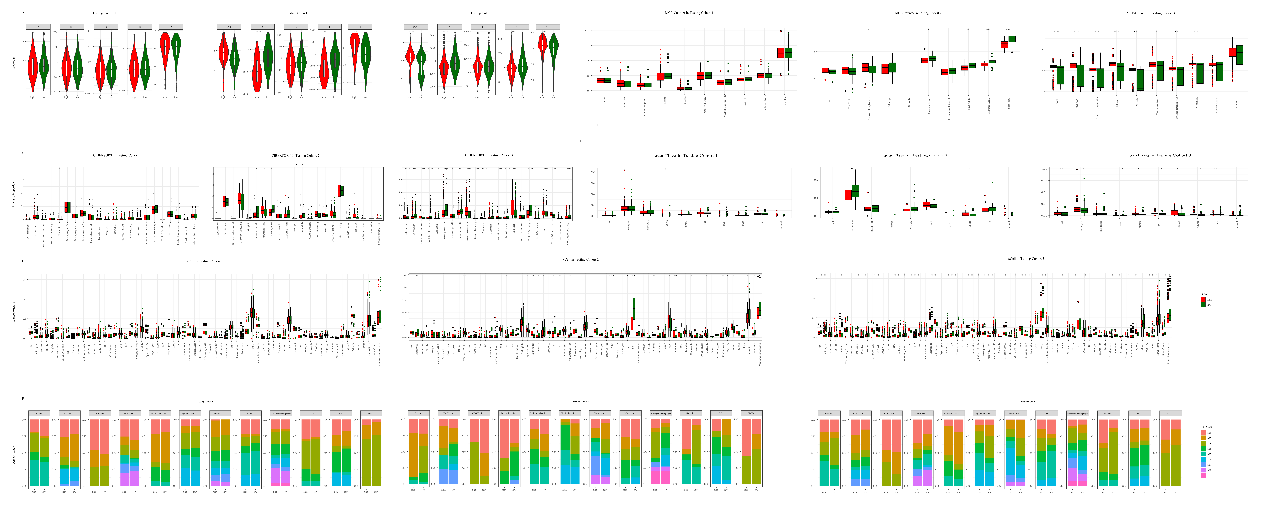


Supplementary Figure 3. Immunological heterogeneity of TMERSS subtypes in the testing cohorts. (A) In the three testing cohorts, CYT, stromal score, immune score, ESTIMATE score and tumor purity were different among TMERSS subtypes; Based on CIBERSORT (B), MCP Counter (C), quanTIseq (D) and xCell (E), the proportion of immune cells between high and low TMERSS subtypes in the three testing cohorts was estimated; (F) Proportional distribution of immune cell states of different TMERSS subtypes in three testing cohorts.


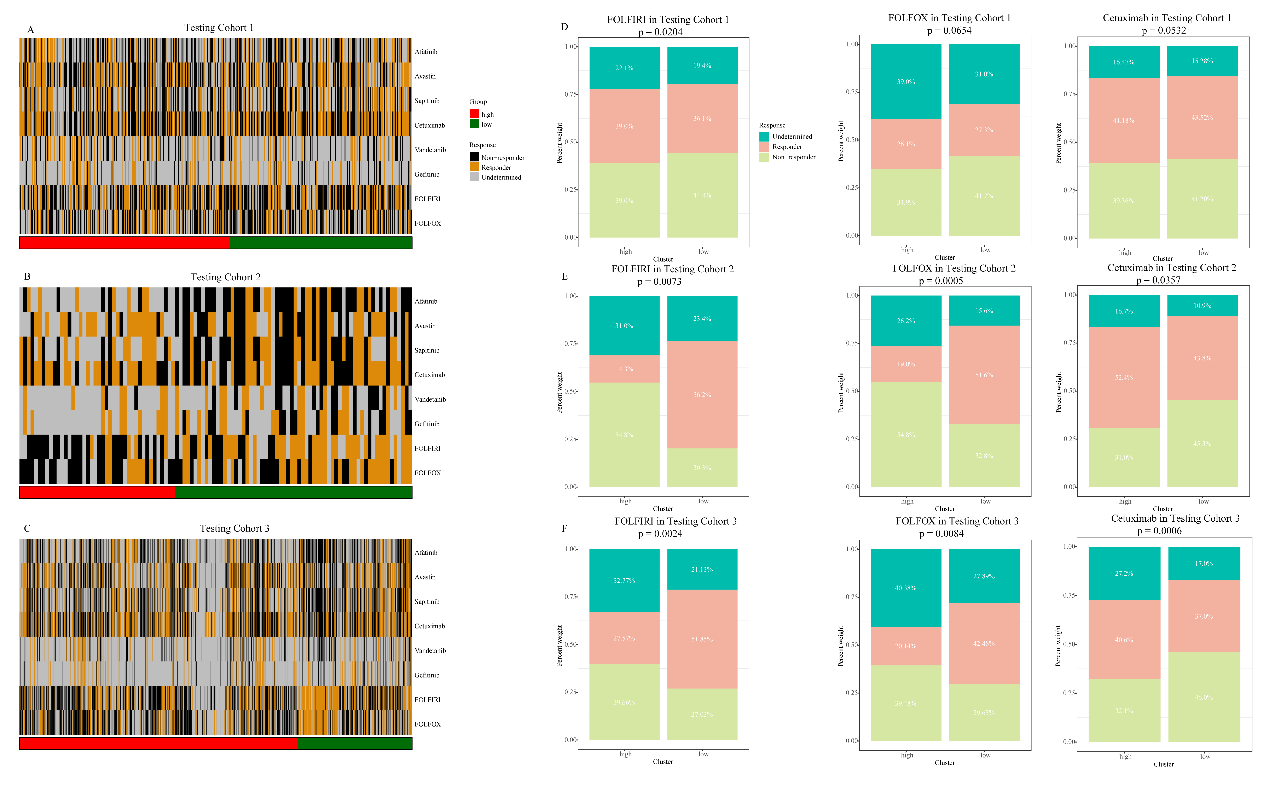


Supplementary Figure 4. To validate the correlation analysis between TMERSS subtypes and chemotherapy in the cohort. (A-C) Heatmaps showing the correlation of responses of single CRC patients in testing cohort 1, 2 and 3 to FOLFIRI, FOLFOX and EGFR inhibitors; The (D-F) histogram shows the number of clinical responses to FOLFIRI, FOLFOX and Cetuximab of high and low TMERSS subtypes in testing cohort 1, 2 and 3, respectively. Chi-square test p-value differences are shown.


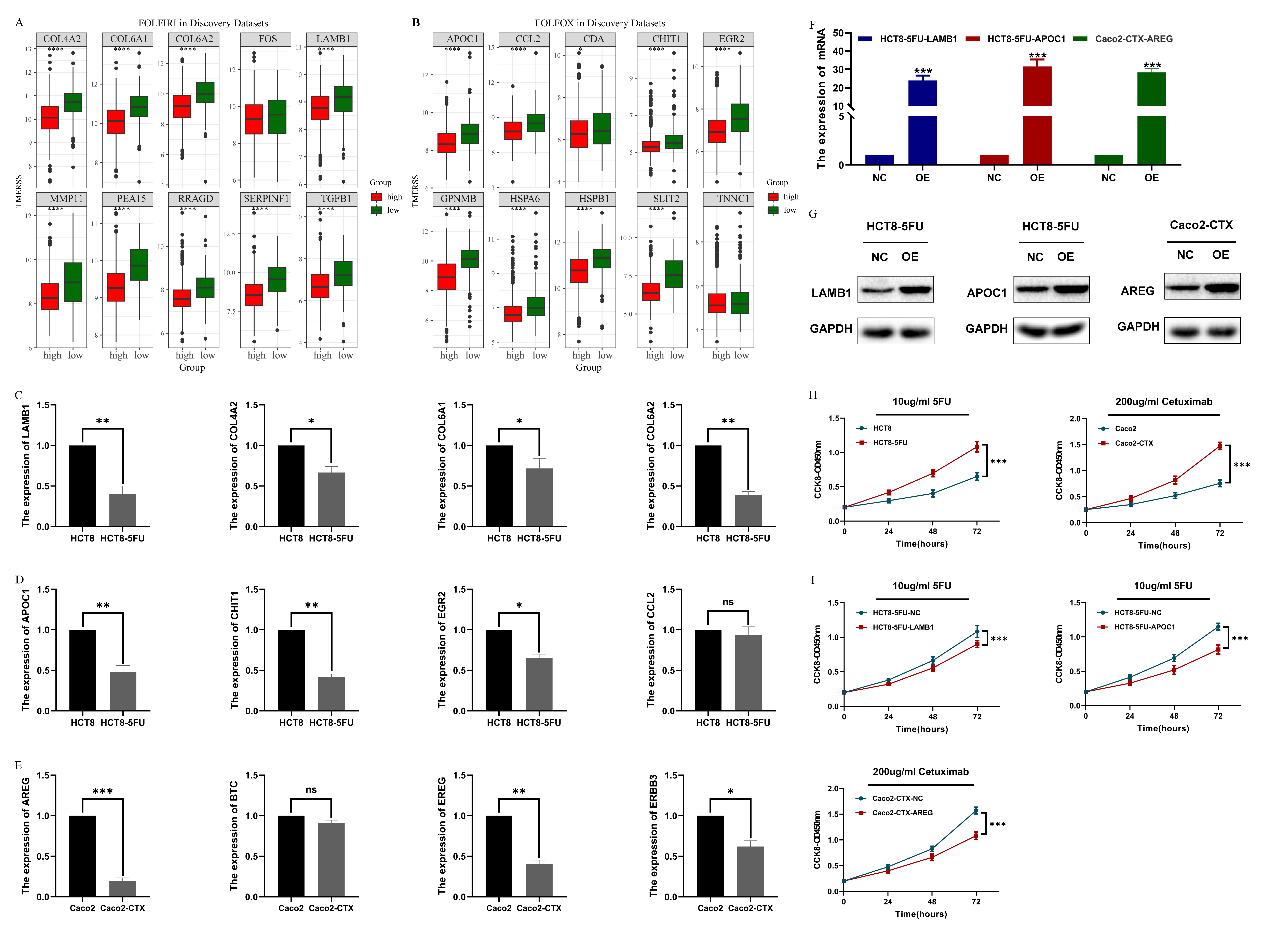


Supplementary Figure 5. Expression analysis of genes related to chemotherapy response. (A-B) The boxplots show the difference in the expression of 5-FU response-related genes in the high and low TMERSS subtypes. (C-E) qRT-PCR was used to detect the relative expression of response-related genes in cell lines. (H) The genes LAMB1, APOC1, and AREG were overexpressed, and then the relative expression levels of LAMB1, APOC1, and AREG in the cell line were detected using qRT-PCR. (G) After overexpressing the genes LAMB1, APOC1, and AREG, western blotting was used to detect the relative protein expression levels of LAMB1, APOC1, and AREG in the cell line. (H-I) CCK8 assay was used to detect the sensitivity of HCT8-5FU/Caco2-CTX cell lines to 5-FU and Cetuximab.
